# Title: *Insects as animal feed: novel ingredients for use in pet, aquaculture and livestock diet*: Editors: Heidi Hall, Elaine Fitches, and Rhonda Smith Publisher: CABI, Oxfordshire, UK (2021), 138 pages ISBN: 9781800620476, paperback

**DOI:** 10.5713/ab.22.0001B

**Published:** 2021-12-23

**Authors:** Beob Gyun Kim

**Affiliations:** 1 Department of Animal Science and Technology, Konkuk University, Seoul 05029, Korea; 2 Animal Bioscience, Seoul 08776, Korea

“Insects as Animal Feed: Novel Ingredients for Use in Pet, Aquaculture and Livestock Diet” edited by Heidi Hall, Elaine Fitches, and Rhonda Smith consists of 16 chapters authored by 50 experts, researchers, and business owners across the world. This book provides recent data on the use of insects as feed ingredients for livestock, pets, fish, and exotics. The authors also discuss the challenges for the future establishment of insect farming as a source of feed ingredients. The readership will include researchers, legislators, insect producers, animal nutritionists, and veterinarians of this area of interest. Although quite a few parts of this book are for the current UK and EU situations, the major topics and issues addressed by the authors would be very helpful for readers worldwide.

**Chapter 1** entitled “The Challenges Facing the Feed Industry” suggests five major criteria to meet for the use of insects as a feed ingredient. Although the authors discuss the situation of the UK mainly, the suggested criteria should be applicable to most other countries.

**Chapter 2** describes the common features of insects reared as a source of animal feed ingredients. Black soldier fly, mealworm, and common house fly are the most largely cultivated as a feed ingredient. Apparently, these insects and potentially other species will become more important as a source of energy and nutrients in animal feeds.

**Chapter 3** describes general processing methods after harvesting black soldier fly larvae. Dried whole larvae, chitin-rich powder, protein powder, and oil are produced depending on the sequential production process. The production processes for functional nutrients including lauric acid rich in black soldier fly, chitin, antimicrobial peptides, and antioxidants are also briefly described. Chemical safety, microbial safety, and allergenicity issues are also discussed in this chapter.

**Chapter 4** summarizes nutrient composition of diverse insect species in the literature. Data for gross energy, proximate analysis, amino acids, and fatty acids are provided in tables. Although the relatively large variation within a specific insect species is one of the challenges to overcome, insect products are a fairly good source of nutrients to animals based on the previous feeding trials. The functional nutrients will add the values of the insect products as feed ingredients.

**Chapter 5 to 8** deal with the beneficial influence of insect farming on other sectors. The additional benefits include recycling of organic wastes to optimize supply chain efficiency, reducing greenhouse gas emissions which is an indicator of global warming potential, and using insect farming byproducts as a fertilizer. The location of the insect farm may be an important factor for these additional benefits.

**Chapters 9 to 12** describe the current status of insect production and utilization of insect products in Asia, Africa, USA and Canada, and Europe. The authors also discuss the challenges for the use of insect products as an animal feed ingredient.

**Chapter 13** is a collection of interviews of 10 entrepreneurs from well-established enterprises to the start-up of pilot phase companies that are AgriProtein (UK), Agroloop (Hungary), Beta Bugs (UK), Beta Hatch (USA), Entec Nutrition (UK), Entocycle (UK), Future Green Solutions (Australia), LIVIN farms (Austria and Hong Kong), nextProtein (France and Tunisie) and Ynsect (France). The experience and opinion of the industry are very helpful for understanding the current status and challenges of the insect production.

**Chapters 14 to 16** provide discussion on the future perspectives on the use of insects as animal feeds. Legislation, policy and quality assurance of many countries are provided and legal limitations on production and use of insect-derived feed materials in the UK and EU are also stated. The acceptance and preference of insects as a feed ingredient by farmers and skate holders in Africa, America, Asia, Australia, and Europe are discussed. In the last chapter, the author emphasizes the use of co-products from insects to increase profitability which is important in most areas in the world.

This book edited by Heidi Hall, Elaine Fitches, and Rhonda Smith is very valuable for discovering recent advances in research and practical applications of insect as a feed ingredient and the challenges that need to be overcome for the safe, economical, and sustainable use of insect products in animal feeds.

